# Association of Chromosomal Alterations with Arsenite-Induced Tumorigenicity of Human HaCaT Keratinocytes in Nude Mice

**DOI:** 10.1289/ehp.7224

**Published:** 2004-07-27

**Authors:** Chia-Wen Chien, Ming-Chang Chiang, I-Ching Ho, Te-Chang Lee

**Affiliations:** ^1^Institute of Biopharmaceutical Science, National Yang Ming University, Taipei, Taiwan, Republic of China; ^2^Department of Life Science, National Central University, Taoyuan, Taiwan, Republic of China; ^3^Institute of Biomedical Sciences, Academia Sinica, Taipei, Taiwan, Republic of China

**Keywords:** arsenite, chromosomal alterations, comparative genomic hybridization, HaCaT cells, tumorigenicity

## Abstract

Inorganic arsenic is a well-documented human carcinogen. Chronic low-dose exposure to inorganic arsenic is associated with an increased incidence of a variety of cancers, including skin, lung, bladder, and liver cancer. Because genetic alterations often occur during cancer development, the objective of this study was to explore what types of genetic alterations were induced by chronic exposure of human HaCaT cells to arsenic. After 20 passages in the presence of inorganic trivalent arsenite at concentrations of 0.5 or 1 μM, HaCaT cells had higher intracellular levels of glutathione, became more resistance to arsenite, and showed an increased frequency of micronuclei. Furthermore, the previously nontumorigenic HaCaT cells became tumorigenic, as shown by subcutaneous injection into Balb/c nude mice. Cell lines derived from the tumors formed by injection of arsenite-exposed HaCaT cells into nude mice expressed higher levels of keratin 6, a proliferation marker of keratinocytes, than did parental HaCaT cells, whereas the expression of keratins 5, 8, and 10 was significantly decreased. Comparative genomic hybridization demonstrated chromosomal alterations in the 11 cell lines derived from these tumors; all 11 showed significant loss of chromosome 9q, and seven showed significant gain of chromosome 4q. The present results show that long-term exposure to low doses of arsenite transformed nontumorigenic human keratinocytes to cells that were tumorigenic in nude mice and that chromosomal alterations were observed in all cell lines established from the tumors.

Arsenic is ubiquitous in nature and is released into the environment via industrial processes and agricultural and medical applications (Chan and Huff 1997). Because of the natural distribution, drinking water is the most common source of arsenic exposure for the general population (Gebel 2000), and millions of people worldwide suffer from arsenic intoxication caused by drinking arsenic-contaminated water (National Research Council 2001). Epidemiologic studies have shown a strong association between chronic arsenic exposure and various adverse health effects, including cardiovascular diseases, neurologic defects, and cancers of the lung, skin, bladder, liver, and kidney (Calderon et al. 2001; Chen et al. 1985, 1995; Chiou et al. 2001; Smith et al. 1992). Although the processes involved in arsenic carcinogenesis remain an enigma, a variety of mechanisms, both genotoxic and nongenotoxic, have been proposed to explain the carcinogenicity of arsenic at the cellular and molecular levels (Kitchin 2001; Rossman 2003).

A risk of arsenic-induced chronic diseases, such as cancer and cardiovascular diseases, is clearly associated with prolonged exposure to low doses of arsenic. Several studies have shown that low doses of inorganic arsenic compounds stimulate the proliferation of mammalian cells (Barchowsky et al. 1999; Germolec et al. 1996; Lee et al. 1985b). Furthermore, long-term exposure to low concentrations of arsenic causes increased neoplastic transformation of murine JB6 Cl41 cells (Huang et al. 1999), blast transformation of human lymphocytes (Meng and Meng 2000), and malignant transformation (tumors formed on injection of arsenic-transformed cells into nude mice) of the rat liver epithelial cell line TRL 1215 (Zhao et al. 1997), the human prostate epithelial cell line RWPE-1 (Achanzar et al. 2002), and the human osteosarcoma cell line TE85 (Mure et al. 2003). Long-term exposure to low doses of arsenite also results in increased tolerance of acute arsenic exposure (Romach et al. 2000) and the aberrant expression of genes involved in the regulation of a variety of cellular functions, including signal transduction, the stress response, apoptosis, and cell proliferation (Chen et al. 2001a, 2001b; Vogt and Rossman 2001). These studies strongly suggest that chronic exposure to low levels of arsenic can produce cellular changes that promote arsenic-induced cell transformation or tumor development.

Over the past few decades, numerous genetic alterations affecting growth-controlling genes have been identified in neoplastic cells, providing persuasive evidence for the genetic basis of human cancer (Lengauer et al. 1998). All tumors contain genetic alterations, including subtle changes in DNA sequences, gene amplification, and gross chromosome losses, gains, translocations, and aneuploidy (Cahill et al. 1999; Schar 2001). Tumors exhibiting abnormal karyotypes involving either chromosomal rearrangement and/or aneuploidy are classified as chromosomal instability tumors (Bardelli et al. 2001). Although arsenic-induced malignant transformation has been shown to be associated with DNA hypomethylation (Zhao et al. 1997), increased matrix metalloproteinase-9 secretion (Achanzar et al. 2002), and delayed mutagenesis (Mure et al. 2003), how arsenic induces genetic and epigenetic alterations during cancer development remains to be elucidated. Treatment with inorganic trivalent arsenite results in the formation of DNA single-strand breaks (Lynn et al. 1997) and in gene amplification (Lee et al. 1988; Yih and Lee 2000). Although inorganic arsenic compounds are ineffective in inducing point mutation in a variety of cultured cell systems (Oberley et al. 1982; Rossman et al. 1980), they cause chromosomal damage in a variety of *in vitro* (Hei et al. 1998; Jha et al. 1992; Lee et al. 1985a) and *in vivo* systems (Gonsebatt et al. 1997). Inorganic arsenic is generally accepted as a clastogenic agent.

We recently reported that treatment with inorganic trivalent arsenite increases the frequency of micronuclei (MN) and aneuploidy in human fibroblasts (Yih et al. 1997). These arsenite-treated human fibroblasts were also shown to have an unstable karyotype but an increased life span (Yih et al. 1997). To explore the association of chromosomal alterations with arsenic-induced tumorigenicity in epithelial cells, an immortalized but nontumorigenic human skin keratinocyte cell line, HaCaT (Boukamp et al. 1988), was exposed to low-dose inorganic trivalent arsenite for a long period. Conversion of the cells from nontumorigenic to tumorigenic was demonstrated by injection of arsenite-exposed cells into nude mice. Chromosomal alterations in the cell lines established from the resulting tumors were analyzed using the comparative genomic hybridization (CGH) technique, which permits the rapid detection and mapping of DNA sequence copy number differences between a normal and an abnormal genome (Kallioniemi et al. 1992). Our results demonstrate that tumor cell lines derived from tumors induced by injection with arsenite-treated cells show chromosomal alterations.

## Materials and Methods

### Cell culture and treatment.

HaCaT cells, kindly provided by N.E. Fusenig (German Cancer Research Center, Heidelberg, Germany), were routinely grown in Dulbecco’s modified Eagle medium (GIBCO, Grand Island, NY, USA) supplemented with 10% fetal bovine serum (GIBCO), 1% glutamine, and antibiotics (100 U/mL penicillin and 100 μg/mL streptomycin) (Boukamp et al. 1988). For long-term exposure of HaCaT cells to arsenite, 5 × 10^5^ cells were plated onto a 100-mm Petri dish and were fed with medium containing various concentrations of sodium arsenite (0, 0.5, and 1 μM). Every 4 days, the cells, grown to near confluence, were subcultured, replated at the same cell density, and fed with arsenite at the same concentration. Subculturing was continued for 20 passages, and the accumulated population doublings during these 20 passages were calculated. HaCaT cells that had been exposed to 0, 0.5, or 1 μM sodium arsenite for 20 passages were designated as A0, A1, or A2 cells, respectively.

### Cytotoxicity assay.

We determined the cytotoxicity of arsenite using the colony-forming assay or the sulforhodamine B (SRB) assay. The colony-forming assay was performed as described previously (Ho and Lee 1999). In brief, the HaCaT cells were treated with various concentrations of sodium arsenite for 24 hr and replated at 200 cells per 60-mm dish in triplicate. Then, after incubation for 10 days, the colonies were fixed, stained, and counted under a dissection microscope. The SRB assay (Skehan et al. 1990) was performed using 96-well microplates and a density of 1,000 HaCaT cells/well. After addition of sodium arsenite, the microplates were incubated for 72 hr, and then the cells were fixed for 1 hr with ice-cold 50% trichloroacetic acid and stained for 30 min with 0.4% (wt/vol) SRB in 1% acetic acid solution. After extensive washes with distilled water, the bound SRB was extracted with 100 μL 10-mM unbuffered Tris-base solution and measured using a 96-well plate reader (Bio-Rad model 550; Bio-Rad, Hercules, CA). The survival curves were plotted by expressing the absorbance of treated wells as a percentage of that of control wells, and the inhibitory concentration 50% (IC_50_) values were calculated by linear regression.

### Glutathione determination.

Cellular glutathione (GSH) levels in logarithmically growing cells were determined as described by Cohn and Lyle (1966).

### Cytokinesis-block MN assay.

We used the method of Fenech and Morley (1989) with slight modifications to analyze the frequency of arsenite-induced MN. In brief, A0, A1, and A2 cells were incubated for 30 hr with 2 μg/mL cytochalasin B and then treated for 150 sec with hypotonic solution (0.05% KCl). After fixation for 8 min in a 20:1 (vol/vol) mixture of methanol and acetic acid, the cells were stained for 10 min with 5% (vol/vol) Giemsa solution, and then the number of MN was scored in 1,000 binucleate cells; under the conditions used, the frequency of binucleate cells was 500–600 per 1,000 cells.

### Tumorigenicity test and establishment of tumor cell lines.

Male Balb/c nude mice 4–6 weeks of age, obtained from the National Laboratory Animal Center (Taipei, Taiwan), were injected subcutaneously with 3 × 10^6^ A0, A1, or A2 cells in 100 μL of phosphate-buffered saline (PBS), pH 7.4, at each of two sites on either side of the back. Five animals were used per cell line and were maintained on regular food and water. To monitor tumor formation, we measured the longest and shortest diameters of the tumors weekly, starting when the tumor was first apparent. At the end of the experiment, the tumors were excised, and then part of the tumor tissue was fixed in buffered formalin for histologic examination, and another part was washed with PBS, minced, digested with collagenase type IV, and seeded in a Petri dish to establish tumor cell lines. Three cell lines, designated T1, T2, and T3, were established from the A1-derived tumors, and two lines, T4 and T5, from A2-derived tumors. To confirm their tumorigenicity, we injected 3 × 10^6^ T1 and T4 cells in 100 μL of PBS into Balb/c nude mice and monitored tumor formation as described above. Two further cell lines, designated T1R1 and T1R2, and four other cell lines, designated T4R1–T4R4, were established from the T1- and T4-induced tumors, respectively. In a separate experiment to see if arsenic enhanced tumor progression, five other cell lines, T4A1–T4A5, were derived from tumors in T4-injected Balb/c nude mice that were also given arsenite-containing water (30–50 ppb) from 1 week before injection until the end of the experiment.

### Western blotting analysis of keratins.

Logarithmically growing cells were scraped from culture dishes using a rubber policeman, lysed immediately in electrophoretic sample buffer, and heated at 95°C for 10 min (Laemmli 1970). Protein concentrations were determined by the Bio-Rad protein assay (Bio-Rad). An aliquot containing 10–20 μg protein was loaded onto a 10% sodium dodecyl sulfate–polyacrylamide gel, and then, after electrophoretic separation, the proteins were transferred onto a polyvinylidene difluoride membrane using a semidry electrotransfer system (ATTO, Tokyo, Japan). After blocking with 5% milk in PBS containing 0.2% Tween 20 for 1 hr at room temperature, the membranes were reacted with primary antibodies against keratin 5/8, 6, 7/17, 10, 14, or 18 (Santa Cruz Biotechnology, Santa Cruz, CA, USA) and horseradish peroxidase–conjugated secondary antibody (Organon Teknika-Cappel, Turnhout, Belgium) as previously described (Yih and Lee 2000). Keratins were then visualized using an enhanced chemiluminescence system according to the manufacturer’s instructions (Pierce, Rockford, IL, USA).

### Chromosomal alteration analysis by CGH.

CGH was performed essentially as described by Kallioniemi et al. (1992) on normal male human lymphocyte metaphase spreads. DNA isolated from control HaCaT cells or cells derived from arsenite-induced tumors was labeled via nick translation with Spectrum red–2′-deoxyuridine 5′-triphosphate (dUTP) and fluorescein isothiocyanate-dUTP, respectively (Vysis, Downers Grove, IL, USA) and the 500–3,000 bp products were used as the probe for CGH. After hybridizing the probe with the spreads for 48–72 hr at 37°C, the slides were washed and counterstained with 4′,6′-diamidino-2′-phenylindole, and then metaphases were examined under a Zeiss Axioskop microscope equipped with appropriate epifluorescence filters and a charge-coupled device camera (SenSys; Photometrics, Tucson, AZ, USA) controlled by the SmartCapture program (Vysis). The filter system (Chroma Technology, Brattleboro, VT, USA) consisted of a triple-bandpass beam splitter and a triple-bandpass computer-controlled filter wheel (Ludl Electronic Products, Hawthorne, NY, USA). Image acquisition, profile generation, and analysis were performed using the Quips XL genetics workstation system (Vysis). After karyotyping, we calculated the green-to-red ratio profiles down the axis of each chromosome. Data from 10 captured metaphases were used to generate a mean profile ± 1 SD per hybridization. Threshold values of 1.2 and 0.8 were set to identify the presence of gains and losses, respectively. To avoid bias due to possible different affinities of the fluorochromes for the DNA, we repeated the hybridization experiment using the same DNA samples from HaCaT cells and arsenite-induced tumor cells, but with the fluorochromes reversed, and used the results from the two hybridizations to determine the gains and losses.

## Results

### Increased intracellular GSH levels and arsenite resistance in long-term arsenite-exposed cells.

**Figure 1:**
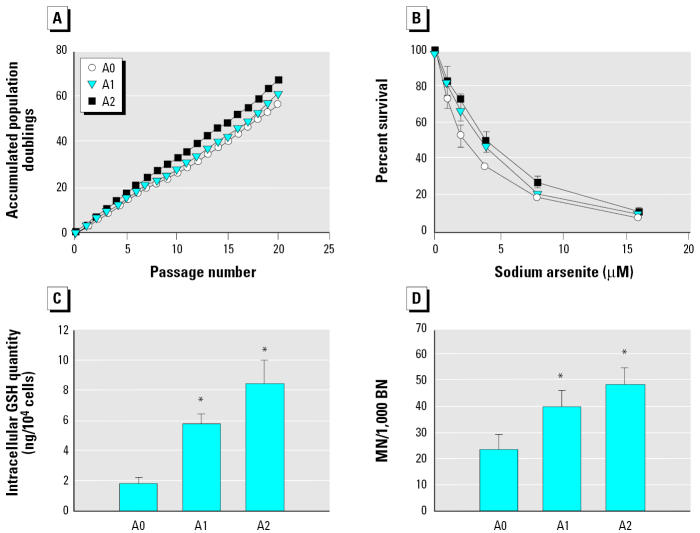
Effects of long-term sodium arsenite treatment on accumulated population doubling, arsenic resistance, intracellular GSH levels, and MN frequency. (*A*) HaCaT cells continuously treated for 20 passages with 0, 0.5, or 1 μM sodium arsenite and then designated as A0, A1, and A2 cells. (*B*) A0, A1, and A2 cells treated with different concentrations of sodium arsenite for 72 hr, and cell survival determined using the SRB assay and the IC_50_ values calculated by linear regression. (*C*) Intracellular GSH levels in A0, A1, and A2 cells. (*D*) MN analysis performed on A0, A1, and A2 cells. In (*B*–*D*), the data are the means ± SD for three independent experiments. *p < 0.05 by Student’s *t*-test (*C*) and by Fisher’s exact test (*D*).

When the colony-forming assay was performed on HaCaT cells treated with arsenite for 24 hr, the value of IC_50_ was 8.7 μM. In a pilot study, 0.5 or 1 μM arsenite did not affect HaCaT cell proliferation. We therefore exposed HaCaT cells continuously for 20 passages to 0, 0.5, or 1 μM arsenite and designated the final cell populations as A0, A1, and A2 cells, respectively. At the doses used, arsenite did not significantly affect the growth rate of HaCaT cells; the accumulated population doublings ranged from 58 to 67 (Figure 1A). However, when the A0, A1, and A2 cells were then exposed to higher concentrations of sodium arsenite (0–16 μM) for 72 hr, the IC_50_ values for arsenite, examined using the SRB assay, were 2.2 ± 0.3, 3.2 ± 0.4, and 3.7 ± 0.5 μM, respectively (Figure 1B). The IC_50_ values for the A1 and A2 cells were significantly higher than that for A0 cells, showing that the A1 and A2 cells were more resistant to arsenite. Consistent with previous reports showing that elevated GSH levels are frequently associated with arsenic resistance (Brambila et al. 2002; Lee et al. 1989), intracellular GSH levels in A1 and A2 cells were significantly higher than those in A0 cells (Figure 1C).

### Increased MN formation in long-term arsenite-exposed cells.

MN, which generally result from the loss of whole chromosomes or chromosome fragments, are frequently used to monitor chromosomal damage and/or instability in *in vitro* and *in vivo* systems (Fenech 2000). We examined the frequency of MN in A0, A1, and A2 cells immediately after exposure to arsenite for 20 passages by using the cytokinesis-block MN technique. As shown in Figure 1D, the frequency of MN in A1 and A2 cells was significantly higher than that in A0 cells, indicating that long-term exposure to low doses of arsenite resulted in increased chromosomal damage.

### Tumorigenicity of HaCaT cells after long-term exposure to a low dose of arsenite.

**Figure 2:**
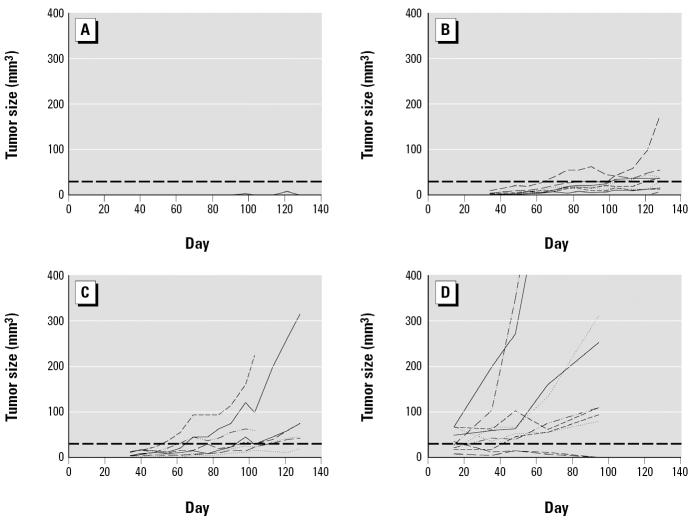
Growth curves of tumors formed in nude mice by subcutaneous injection of A0, A1, A2, or T4 cells. Tumor size (longest × shortest^2^ diameter × 0.5 in mm^3^), measured once a week starting 1 month (A0–A2 cells) or 2 weeks (T4 cells) after injection, is plotted against time. (*A*) A0 cells. (*B*) A1 cells. (*C*) A2 cells. (*D*) T4 cells. The horizontal lines indicate 30 mm^3^, and a tumor size greater than this was considered tumor formation in Table 1.

**Figure 3:**
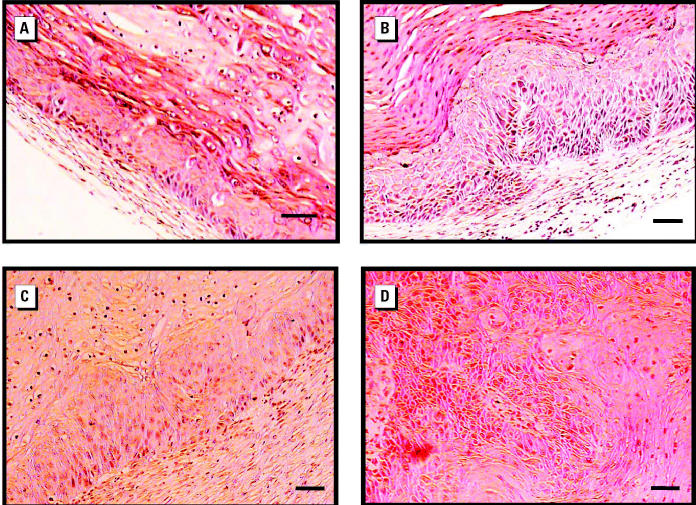
Histologic examination of tumors formed in nude mice. Tumors showing a hyperplastic stratified epithelium with prominent parakeratosis formed by injection of A1 cells (*A*) or A2 cells (*B*). Tumors with more malignant characteristics formed by injection of cell lines T1 (*C*) or T4 (*D*). Bars = 50 μm.

We examined the tumorigenicity of A0, A1, and A2 cells by injecting the cells into Balb/c nude mice. As shown in Figure 2, no tumor growth was seen after injection of A0 cells, whereas tumors were seen 2 months after injection of A1 or A2 cells. As summarized in Table 1, tumors were formed at five or seven of the 10 sites injected with A1 or A2 cells, respectively. Histologic examination of the tumors revealed the formation of a multilayered, hyperproliferative, keratinizing epithelium (Figure 3A,B). When two tumor cell lines, T1 and T4—derived, respectively, from tumors induced by injection with A1 or A2 cells—were reinjected into nude mice to confirm their tumorigenicity, tumors were rapidly formed within 2 weeks at almost all injection sites (Figure 2D, Table 1). Their histologic phenotypes were clearly more malignant than those formed after injection with A1 or A2 cells (Figure 3C,D). When T4 cells were injected into nude mice given arsenite-containing water from 1 week before injection until the end of the experiment, the number of tumors formed and the rate of tumor formation were the same as in similarly injected nude mice given arsenite-free water (data not shown), showing that the continued presence of arsenite did not enhance tumor progression.

### Altered keratin expression in long-term arsenite-exposed cells and cell lines derived from arsenite-induced tumors.

**Figure 4:**
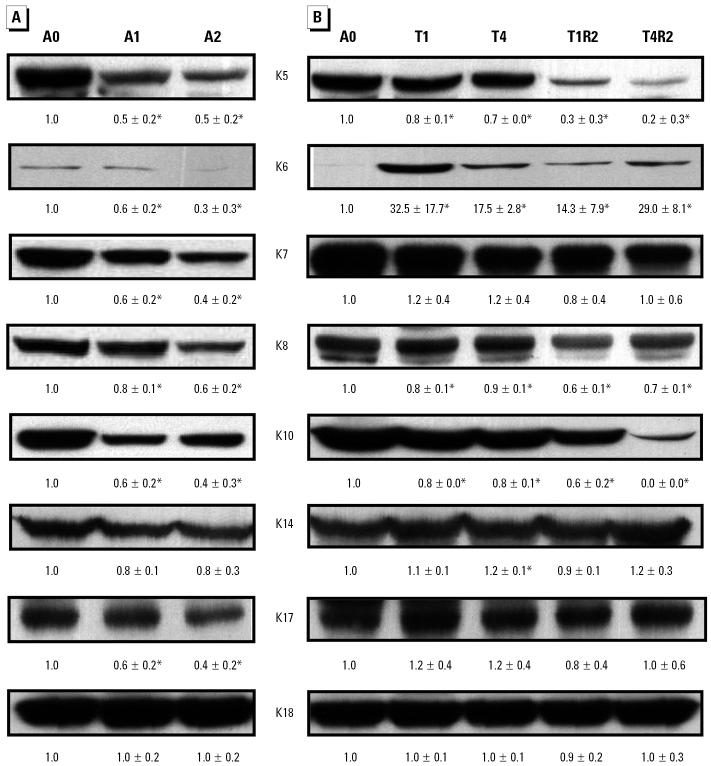
Western blotting analysis of keratins in arsenite-exposed cells and cell lines derived from tumors induced by injection of arsenite-treated cells. (*A*) Keratin levels in A0, A1, and A2 cells. (*B*) Keratin levels in cell lines derived either from tumors induced by injection of arsenite-treated cells (T1 and T4) or from those induced by injection of lines T1 or T4 (T1R2 and T4R2). β-Actin was used as the loading control and to normalize the keratin expression levels. The normalized expression level in each cell type was then compared with that in A0 cells. The data are the means ± SD for three independent experiments. *p < 0.05 by Student’s *t*-test.

Keratins are components of intermediate filaments and play an essential role in cytoskeleton formation (Morley and Lane 1994). They are involved in a variety of cell functions, and alterations in keratin expression are closely associated with tumor progression (Chu and Weiss 2002). With Western blotting, the levels of keratins 5, 6, 7, 8, 10, and 17 were significantly decreased in A1 and A2 cells compared with A0 cells (Figure 4A), whereas levels of keratins 14 and 18 remained relatively constant. A significant decrease in levels of keratins 5, 8, and 10 was also observed in all cell lines established from tumors (Figure 4B). The expression of these keratins in T1R2 and T4R2 cells was in general lower than that in the parental T1 and T4 cells. The levels of keratins 7, 14, 17, and 18 did not change in these cell lines, whereas, because of the very low levels in A0 cells, the levels of keratin 6, a proliferation marker, were markedly increased (Figure 4B).

### Identification of chromosomal alterations in cell lines derived from long-term arsenite-exposed cells.

**Figure 5:**
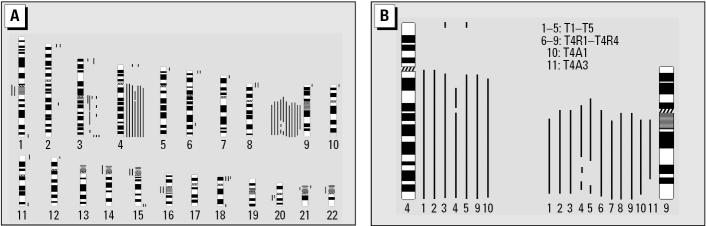
CGH analysis of cell lines derived from arsenite-induced tumors. (*A*) All cell lines were compared with HaCaT cells. Chromosomal loss is indicated by a line to the left of each chromosome, and a gain by a line to the right. The entire X and Y chromosomes were excluded from analysis. (*B*) Chromosomal gain at the 4q region and loss at the 9q region in individual cell lines. Lines T1–T5 were derived from tumors induced in mice injected with A1 or A2 cells, whereas the other six lines were from tumors induced by injection of T4 cells.

To evaluate the presence of genetic changes in arsenite-induced tumors, we performed CGH analysis to analyze DNA sequence copy number changes in cell lines derived from tumors produced by injection with A1 or A2 cells or cell lines derived from the resulting tumors. The major changes found in these cell lines were gain of chromosome 4q and loss of chromosome 9q (Figure 5A). Other regions occasionally showing gain and loss of chromosome regions are summarized in Figure 5A. In a detailed comparison (Figure 5B), all five tumor cell lines established from A1 and A2 cells (lines T1–T5) showed gain of chromosome 4q and loss of a large region of chromosome 9q. However, although all six of the cell lines derived from tumors formed by injection with T4 cells showed loss of chromosome 9q, only two (lines T4R4 and T4A1) showed gain of chromosome 4q. These results show that 9q12–22 was lost in all these cell lines and that chromosomal alteration, particularly loss of chromosome 9q, was a common event in tumor cells derived from arsenite-exposed HaCaT cells.

## Discussion

Chronic arsenic exposure results in skin pathology, including hyperkeratosis, pigmentation changes, Bowen’s disease, basal cell carcinomas, and squamous cell carcinomas (Centeno et al. 2002). In the present study, we demonstrated that long-term low-dose exposure to sodium arsenite converted the nontumorigenic human keratinocyte HaCaT cell line into cells that were tumorigenic in nude mice. Histology of the tumors caused by injection of arsenite-treated HaCaT cells showed epithelial hyperplasia, mild dysplasia, severe dysplasia, and invasive carcinoma. These phenotypes are similar to arsenic-induced skin pathology. These results showing the induction of neoplastic transformation by long-term exposure of nontumorigenic cells to low doses of arsenite are consistent with those of several other studies using different cell systems (Achanzar et al. 2002; Huang et al. 1999; Mure et al. 2003; Zhao et al. 1997).

Consistent with several previous reports (Brambila et al. 2002; Lee et al. 1989; Romach et al. 2000), we showed that long-term exposure of HaCaT cells to low doses of arsenite resulted in an increase in intracellular GSH levels and resistance to arsenite challenge. These results also suggested that the insults produced by low-dose arsenite stress modulated the cellular biochemistry to adapt to the growth environment. Because acquisition of a survival advantage is crucial for the development of cancer (Hanahan and Weinberg 2000), long-term exposure to arsenite, even at low doses, warrants concern.

In *in vitro* systems, arsenite induces MN in a variety of cells (Eastmond and Tucker 1989; Liu and Huang 1997; Wang and Huang 1994). Both low-dose and high-dose exposure to arsenite induces MN formation (Yih and Lee 1999), but low-dose treatment results mainly in kinetochore-positive (K^+^) MN, whereas high-dose treatment results mainly in K-negative MN. K^+^ MN are usually caused by failure of the whole chromosome to segregate into daughter cells, and agents inducing aneuploidy by interfering with spindle formation often induce K^+^ MN formation (Eastmond and Tucker 1989). Thus, low-dose arsenite may be considered an aneugen. In fact, an increased frequency of MN has been demonstrated in exfoliated bladder cells, buccal cells, sputum cells, and lymphocytes from arsenic-exposed populations (Rossman 2003). The increased frequency of MN seen in A1 and A2 cells in this study shows that long-term exposure to low-dose arsenite can cause chromosomal damage. Because chromosomal alterations are a general manifestation of tumors (Cahill etal. 1999; Schar 2001), the effects of arsenic-induced chromosomal damage may play a role in arsenic tumorigenesis.

Keratins are the major structural proteins in epithelial cells and consist of a family of proteins (Morley and Lane 1994). Several human genetic diseases provide evidence that keratins function to protect cells from mechanical and nonmechanical stresses that result in cell death (Fuchs and Cleveland 1998; Ma et al. 2001). The expression of keratins is affected by cellular differentiation, environmental stimuli, and diseases (Morley and Lane 1994). Progressive alterations in keratin expression are closely associated with the development of a variety of tumors (Chu and Weiss 2002). In our present study, long-term exposure of HaCaT cells to low-dose arsenite caused a reduction in the levels of keratins 5, 6, 7, 8, 10, and 17, and the cell lines derived from tumors induced by injection with arsenite-treated cells had a similar pattern of expression of keratins, except that the levels of keratins 7 and 17 were unchanged and keratin 6 levels were significantly increased in the tumor cell lines. These results show that long-term arsenite exposure can alter regulation of keratin expression. Levels of keratin 6, a marker of hyperproliferative keratinocytes (Tomic-Canic et al. 1998), are increased during wound healing, psoriasis, and other inflammatory disorders (Tomic-Canic et al. 1998). Furthermore, increased levels of keratins 6 and 16 have been reported in arsenic-induced Bowen’s disease, and increased levels of keratins 6, 16, and 17 are seen in arsenic-induced squamous cell carcinoma and basal cell carcinoma (Yu et al. 1993). The increased keratin 6 expression seen in tumor cell lines derived from long-term arsenite-exposed HaCaT cells suggests that keratin 6 is a good proliferation marker for arsenite-induced carcinogenesis.

Using CGH analysis, we demonstrated genetic changes in cells exposed to low-dose arsenite for a long time. Because gain of chromosome 4q and loss of 9q were observed in most of the cell lines established, these non-random changes are possibly important genetic events in arsenic tumorigenesis. However, although gain of chromosome 4q was seen in all five lines cells derived from A1- and A2-induced primary tumors (lines T1–T5), it was only seen in two (T4R4 and T4A1) of six cell lines derived from T4-induced secondary tumors. This suggests that gain of chromosome 4q might not be crucial for arsenite-induced tumorigenicity. On the other hand, loss of chromosome 9q was consistently observed in all primary and secondary tumor cell lines established in this study, suggesting that it plays an essential role in arsenite-induced tumorigenicity. Deletion of all or part of chromosome 9q is seen in tumors from patients exposed to arsenic (Moore et al. 2002). As reported by Boukamp et al. (1997), HaCaT cells are spontaneously immortalized human skin keratinocytes and remain nontumorigenic up to 300 passages (Boukamp et al. 1997). Because translocations and deletions occurred during late passages, the presence of rare tumorigenic variants in A0 cells warrants our concern. However, it is unlikely because the sustained nontumorigenic phenotype of HaCaT cells during long-term propagation is well associated with their preserved chromosomal balance demonstrated by karyotypic and CGH analysis (Boukamp et al. 1997).

The association of chromosomal alterations with cancer development is a complicated issue. Gain of chromosome 4q or loss of 9q has been found in a variety of cancers, including skin, bladder, and lung cancers (Hartmann et al. 2002; Merlo et al. 1994; Popp et al. 2000), but other studies found an association between loss of chromosome 4q or gain of 9q and cancer development (Balsara et al. 2001; Jin et al. 2001). These studies indicate the presence of both tumor suppressor genes and oncogenes on these chromosomal regions. The genes for chemokine ligands 1, 2, and 3 are localized on chromosome 4q (Haskill et al. 1990) and are considered oncogenes because of their growth stimulatory activity. Two putative tumor suppressor genes, deleted in bladder cancer 1 (*DBC1*) and deleted in esophageal cancer 1 (*DEC1*), are localized on chromosomal 9q. Loss of heterozygosity of *DBC1* is seen in some bladder cancers (Habuchi et al. 1998), whereas *DEC1* expression is reduced or absent in esophageal squamous cell carcinomas (Nishiwaki et al. 2000). The expression of these genes and its relationship to arsenic carcinogenesis require further investigation.

In conclusion, our results demonstrate that long-term exposure to low doses of arsenite can cause genetic instability and lead to conversion of nontumorigenic human epithelial cells into cells that are tumorigenic in nude mice. However, the oncogenes and/or tumor suppressor genes involved in arsenic-induced carcinogenesis require further investigation.

## Figures and Tables

**Table 1:** Tumorigenicity of arsenite-exposed cells and cell lines derived from arsenite-induced tumors after subcutaneous injection into nude mice.

Cells*^a^*	Days	No. of tumors/no. of injections (%)	Tumor size (mm^3^)*^b^*
A0	128	0/10 (0)	
A1		5/10 (50)	68.3 (36.5–174.1)
A2		7/10 (70)	119.0 (42.1–314.1)
T1	35	4/4 (100)	146.5 (105.4–185.6)
T4	94	8/10 (80)	413.2 (78.9–1242.8)

a A0, A1, and A2, final cell lines after treatment with 0, 0.5, or 1 μM sodium arsenite, respectively, for 20 passages; T1 and T4, cell lines derived from tumors induced by injection with A1 cells or A2 cells, respectively.

b Tumor size = longest × shortest2 diameter (in mm) × 0.5.
